# Stress Concentration Factors of Concrete-Filled T-Joints under In-Plane Bending: Experiments, FE Analysis and Formulae

**DOI:** 10.3390/ma15186421

**Published:** 2022-09-16

**Authors:** Feleb Matti, Fidelis Mashiri

**Affiliations:** School of Engineering, Western Sydney University, Second Ave., Kingswood, NSW 2747, Australia

**Keywords:** concrete-filled chords, design equations, in-plane bending, SHS T-joints, stress concentration factor

## Abstract

Experimental and numerical investigations on seven cold-formed steel square hollow section (SHS) T-joints with concrete-filled chords were conducted for the determination of stress concentration factors (SCFs). The SCFs were experimentally determined using strain gauges and then numerically determined using Abaqus finite element analysis (FEA) software under static in-plane brace bending. Good agreement was observed between the two investigations. After validating the FEA results, a parametric study was conducted on the SCFs of concrete-filled SHS-SHS T-joints using Abaqus FEA to evaluate the effects of the non-dimensional parameters on the SCFs. Subsequently, design formulae for predicting the SCF of concrete-filled SHS-SHS T-joints subjected to in-plane bending were proposed. Comparable results were obtained between the numerical SCFs with SCFs calculated from the proposed design equations. The maximum SCF of concrete-filled SHS T-joints under in-plane brace bending occurred at different locations. The overall mean of the experimental reduction percentage in peak SCF due to concrete infill is 22% and the overall mean of the numerical reduction percentage in peak SCF due to concrete infill is 19%. The determination of SCFs in concrete-filled SHS-SHS T-joints under in-plane bending has been the subject of little research, and more information regarding the behavior of concrete-filled T-joints with SHS under in-plane bending needs to be provided to practicing engineers.

## 1. Introduction

Cold-formed welded tubular T-joints made up of square hollow sections (SHSs) are extensively used in onshore and offshore structures such as bridges, cranes, and towers. This is because SHS T-joints provide a higher strength-to-weight ratio compared to conventional sections and are easily fabricated and welded due to their flat faces, which are cost and time effective. The SHS T-joints used in engineering structures are generally subjected to cyclic loading such as repeated in-plane bending from permanent loads, for example, the weight of a deck located on top of a truss, or imposed loads such as vehicles and wind loads. When cyclic loads are applied to a structure, initiation and propagation of cracks at the hot spot locations and subsequent fatigue failure may occur. Therefore, filling the chord of the SHS T-joints with concrete generally reduces the SCFs and improves the fatigue behavior of joints as concrete infill prevents inward buckling.

Previous research on SHS joints under in-plane bending was carried out by [1,2,3,4,5]. CIDECT Design Guide 8 [6] proposed SCF equations for empty SHS-SHS T-joint connections under in-plane brace bending but it does not provide SCF equations for concrete-filled SHS-SHS T-joint connections under in-plane brace bending. Ref. [7] conducted experimental and numerical investigations on SCFs of empty SHS-SHS T-joints under static in-plane brace bending and focused on reporting the experimental investigation and model validation. Results showed a similar trend of the variation of SCF with the non-dimensional parameter (β) from experiments and numerical analysis. In addition, previous research on circular hollow section (CHS) joints under in-plane bending were carried out by [8,9,10]. These researchers proved that the concrete filling of the chord joints generally reduced the SCFs at the hot spot locations.

In this paper, more SCF results at the hot spot locations of SHS-SHS concrete-filled T-joint connections will be reported to provide more information on the behavior of concrete-filled SHS T-joint specimens and enable the authors to propose design formulae. This is achieved by testing T-joints with smaller β values (β < 0.35) and larger β values (β > 0.67) than those previously reported by [1]. Currently, there are no design formulae for calculating the SCFs at the hot spot locations of cold-formed welded tubular SHS-SHS T-joints with concrete-filled chords under in-plane brace bending. This is due to limited results relating to the concrete grades, size range, and non-dimensional parameters of hollow structural steel sections and joint types. 

The current paper focuses on parametric studies and the determination of design graphs and predictive equations for determining the SCFs in SHS-SHS T-joints with concrete-filled chords under in-plane brace bending. Therefore, this paper provides new results on SCFs of concrete-filled SHS T-joints under in-plane bending which will contribute to the fatigue design and performance evaluation of existing and new tubular structures to prevent possible collapse or fatigue failure of structures with concrete-filled SHS-SHS T-joints. Another benefit of this research is that the design formulae proposed in this paper will allow researchers and engineers to easily determine the life of welded composite tubular structures. This will save time and cost as experimental and numerical investigations will not be required to evaluate the life of welded composite tubular structures. Finite element modeling of concrete-filled SHS-SHS T-joints using Abaqus software provides a low-cost solution within a relatively short time [11] and human error in the experimental lab is prevented because the study is conducted in a computer program.

## 2. Experimental Program

### 2.1. Specimens and Material Properties

A total of seven cold-formed welded tubular SHS-SHS T-joints, listed in Table 1, were tested experimentally to determine the stress concentration factor (SCF) at the hot spot locations and to validate the FE results. Each T-joint specimen is made up of an empty SHS brace welded to a concrete-filled SHS chord. As shown in Table 1, the non-dimensional parameters of the 7 SHS-SHS concrete-filled SHS T-joints are as follows: 0.25≤β≤1; 25.00≤2γ≤33.3; and 0.75≤τ≤1. For each T-joint connection, β was calculated using the ratio of the brace width and the chord width (b_1_/b_0_), 2γ was calculated using the ratio of the chord width to chord wall thickness (b_0_/t_0_), and τ was calculated using the ratio of brace wall thickness and the chord wall thickness (t_1_/t_0_). The brace and chord lengths of each specimen are 500 mm and 600 mm, respectively. The T-joint specimens are made up of a SHS brace welded to a concrete-filled SHS chord. The dimensions of the weld were determined in accordance to AS 4100-1998 [12]. The weld leg length is 6 mm, and the weld throat thickness is 4.24 mm. The nominal tensile strength of the weld metal is 480 MPa, in accordance with AS 4100-1998 [12]. Since the non-dimensional parameters of the SHS T-joints are the same as other research by [4,7], further details about the specimens can be found in their papers. Ref. [4] conducted tensile coupon tests and found that the average value of the Young’s Modulus of the steel SHSs is approximately 200,000 MPa; Ref. [4] also found that the average values of the yield stress are greater than the specified minimum yield stress of 350 MPa. The average values of the ultimate tensile strength are also greater than the minimum specified ultimate tensile strength of 430 MPa. Concrete cylinder tests were carried out to determine the compressive strength of the concrete at 14, 28, 42, 70, and 77 days. Before applying the compression loads to the concrete cylinders, the concrete cylinders’ finished end was ground AS 1012.9–2014 [13]. Three concrete cylinder tests were conducted to determine the 28-day compressive strength. The compressive strength of the concrete after 28 days was 33.17 MPa, 35.40 MPa, and 43.04 MPa. The average compressive strength of the concrete increases after 28 days and is greater than 37 MPa.

### 2.2. Instrumentation, Test Setup, and Loading

**Figure 1:**
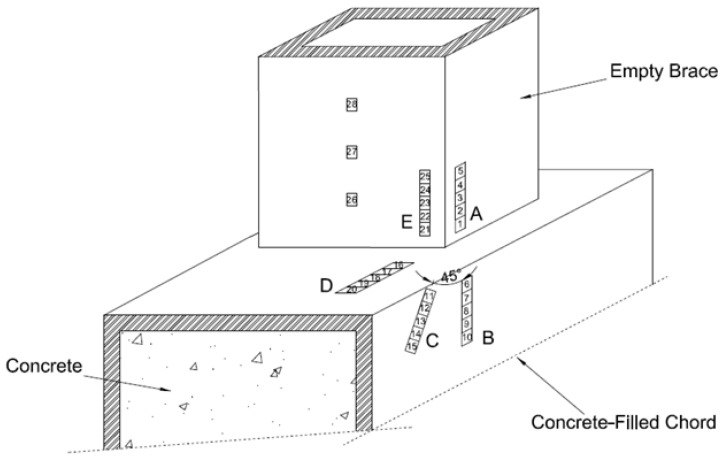
Locations of strain gauges.

**Figure 2:**
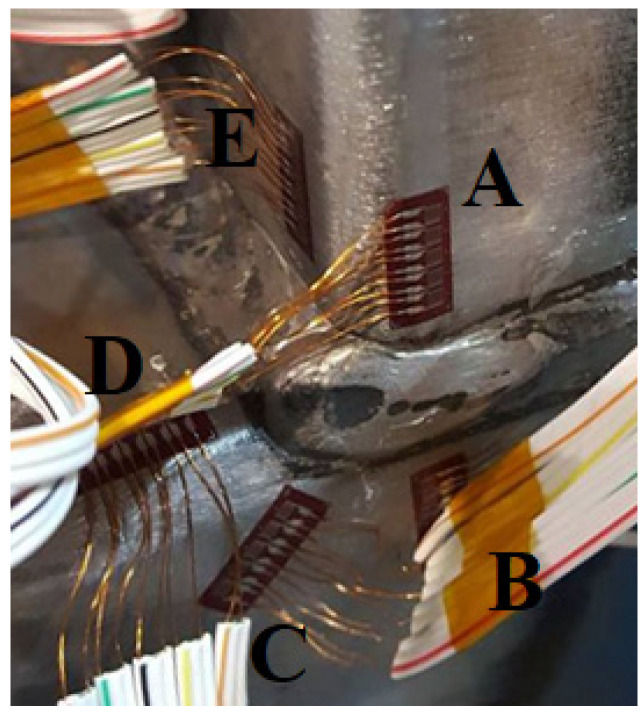
Hot spot locations.

As shown in Figure 1, each T-joint specimen consisted of three single strain gauges installed on the tension side of the brace. Five strip strain gauges were installed on the brace-chord intersection as shown in Figure 2. The strip strain gauge is a parallel-axis strip gauge. The strip strain gauges comprise 5-element single strain gauges which are 2 mm apart. Strip strain gauges were installed at the hot spot locations (lines A–E). As recommended by CIDECT Design Guide 8 [6]; the distance of the strain gauge from the weld toe is at least 0.4 t or 4 mm but a minimum of 4 mm.

**Figure 3:**
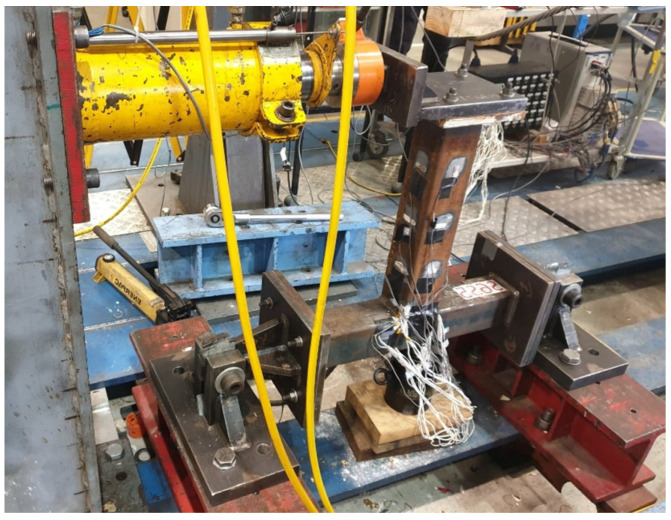
Test setup.

The T-joint specimens were connected to the test rig through grade 8.8 M12 bolts under in-plane brace bending, as shown in Figure 3. The test setup was used to measure the strain distribution. The end plates were bolted to the end brackets to support the T-joint connection. The test rigs used for the experiments are from Construction Technology Laboratory Western Sydney University, a NATA accredited Laboratory with connection testing capabilities in compliance with Australian and ISO standards. All the equipment was calibrated through reliable test results. In-plane bending moment loads were applied to each specimen’s brace in the direction of the longitudinal axis of the chord. The loads applied to each concrete-filled T-joint are within the elastic response range of the load-deformation curve of the connection. Post-elastic behavior was ignored to allow the researcher to carry out the fatigue testing correctly, as exceeding the plastic limit load will result in specimen failure. The increased values of the in-plane bending loads applied to each specimen are shown in Table 4.

### 2.3. Experimental SCF

**Figure 4:**
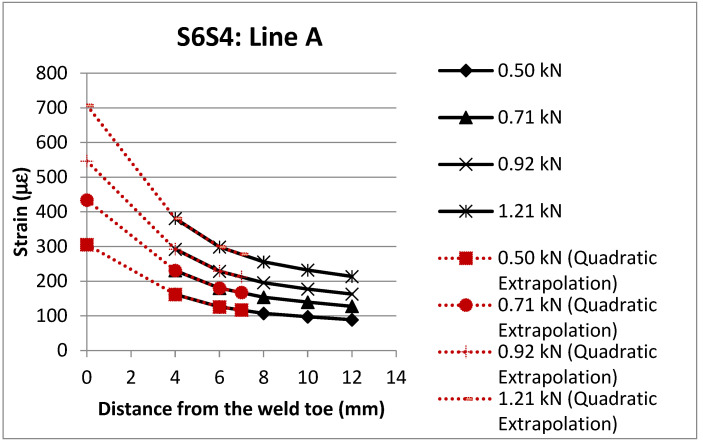
Determination of hot spot strains using quadratic extrapolation.

The experimental SCFs were determined using the ratio of the hot spot strain and nominal strain, as expressed by Equation (1). As recommended by CIDECT Design Guide 8 [6], hot spot strains were determined using the quadratic extrapolation method; see Figure 4 for a typical example of this method for determining the hot spot strains of specimen S6S4 along line A. Hot spot strains were calculated using the strip strain gauges on the chord member. The nominal strains were calculated by linearly extrapolating the measured strains using the single strain gauges on the brace member; see Figure 1 for the extrapolation points. The ratio of the hot spot strain and the nominal strain expressed in Equation (1) is multiplied by the safety factor of 1.1, as recommended by CIDECT Design Guide 8 [6]. Table 2 shows the values of the nominal strains for specimen S6S4.

(1) $${\mathrm{SCF}}_{\mathrm{Test}}=1.1\times \frac{\mathrm{Hot}\;\mathrm{Spot}\;\mathrm{Strain}\;\left(\mathrm{HSSN}\right)}{\mathrm{Nominal}\;\mathrm{Strain}}$$

To validate the experimental nominal strains, the experimental nominal strains were converted to experimental nominal stresses. In addition, nominal stresses using simple beam theory were determined using the ratio of the bending moment and the elastic section modulus of the brace. Table 3 shows that there is a good agreement between the experimental nominal stresses and the nominal stresses from simple beam theory. The experimental SCFs along lines A–E of the seven concrete-filled SHS-SHS T-joint specimens under in-plane brace bending are shown in Table 4.

## 3. Finite Element Analysis (FEA)

### 3.1. General

Finite element analysis was carried out using Abaqus software to capture the distribution of the numerical SCFs of welded tubular SHS-SHS T-joints with concrete-filled chords. Three-dimensional (3D) 8-noded liner hexahedral brick elements with reduced integration (C3D8R) were used to model sixty (60) SHS-SHS concrete-filled T-joints under in-plane bending. The 3D 8-node hexahedral solid finite element is widely used for the FEA of solids in engineering practice. The element can be used to model many three-dimensional solids and performs considerably better than the 4-node tetrahedral element [14]. Ref. [15] stated that eight-node hexahedrons are known as bricks, which lead to more accurate results and reliable FEA solutions. Meshes consisting of hexahedrons are easier to visualize than meshes consisting of tetrahedrons, and the reaction of hexahedral elements to the application of body loads more precisely corresponds to loads under real-world conditions. In total, 7 T-joints identical to the experimental T-joints were created and a further 54 SHS-SHS T-joints with concrete-filled chords were modeled and analyzed under in-plane brace bending. The non-dimensional parameters of the 60 concrete-filled SHS-SHS T-joints are as follows: 0.25≤β≤1; 16.67≤2γ≤33.3; and 0.4≤τ≤1.

### 3.2. Material Properties, Interaction, and Loading

**Figure 5:**
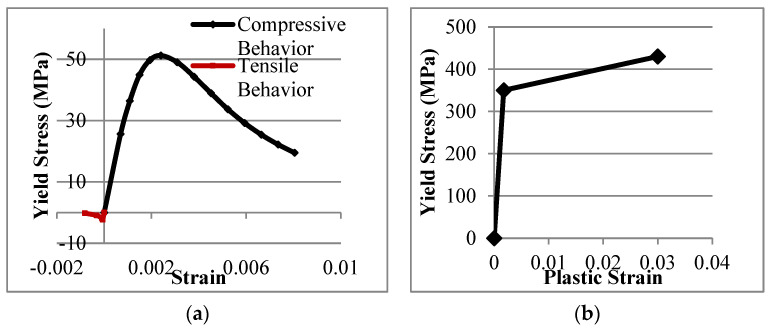
Stress−strain relationship used in the FEA simulation: (**a**) Concrete and (**b**) Steel.

**Figure 6:**
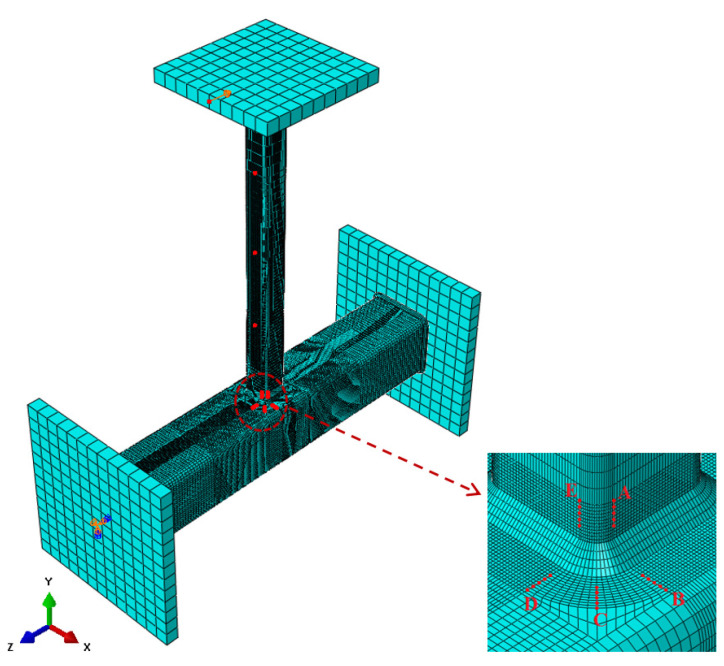
Hot spot locations of a typical FE mesh model.

The design yield stress, tensile strength, Young’s Modulus, and Poisson’s ratio incorporated in the FEA are 350 MPa, 430 MPa, 200 GPa, and 0.3, respectively. The stress-strain distribution of both concrete and steel is displayed in Figure 5. The use of design values for the material properties produced results that were comparable to the experimental SCFs. The SCFs and the structural durability of the structures depend on the boundary conditions, welding size, loading cases, and geometry of the joints [16,17,18]. Ref. [19] also stated that the distribution of the SCF is dependent on the geometrical parameters of the joints. The brace-chord intersection was tied with the weld. For the interaction between the concrete and the SHS steel chord, the coefficient of friction between concrete and steel is 0.35 until the two surfaces are separated. This value was adopted based on previous research by [20,21,22]. The interface element allows the contact surfaces to slide and separate when the load is applied. A static in-plane bending load of 0.60 kN was applied to each of the 60 T-joint models using the Static, General procedure available in the ABAQUS library. The hot spot locations and the direction of the in-plane bending load in a typical meshed model are shown in Figure 6. Mesh convergence analysis was carried out by [2], and the details of the mesh size are reported by [4]. A finer element edge was used in the region around the chord-brace intersection; 1 mm in length at the locations of interest (lines A–E) or less than 1 mm along lines B and C for β = 1.

### 3.3. Numerical SCF

The numerical SCFs at the hot spot locations (lines A–E) are determined using the ratio of the hot spot stress and the nominal stress as expressed in Equation (2).

(2) $${\mathrm{SCF}}_{\mathrm{FEA}}=\frac{\mathrm{hot}\;\mathrm{spot}\;\mathrm{stress}\;}{\mathrm{nominal}\;\mathrm{stress}\;}$$

**Figure 7:**
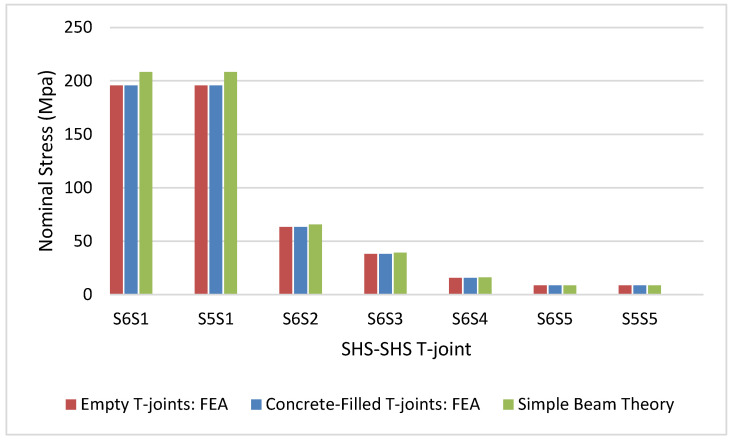
Nominal stresses.

Quadratic extrapolation was used to calculate the hot spot stresses and the linear extrapolation method was used to determine the nominal stresses. Nominal stresses were also determined using simple beam theory which expresses nominal stress as the ratio of the bending moment and the elastic section modulus of the brace. Table 5 and Figure 7 show that the numerical nominal stresses agree with the values determined from the simple beam theory. The nominal stresses of the T-joints with concrete-filled chords are the same as those for the corresponding empty T-joints reported by [7]. Table 6 summarizes the process for determining the SCFs from FEA of concrete-filled SHS-SHS T-joints under in-plane brace bending.

## 4. Results

### 4.1. SCFs

The experimental SCFs of the seven concrete-filled SHS-SHS T-joint under in-plane bending are shown in Table 7. The SCFs of the T-joints with concrete-filled chords were compared with the empty T-joints reported by [7]. The peak experimental SCFs of the empty and concrete-filled SHS T-joints occurred on the cord, with β ranging from 0.25 to 0.50. The peak SCF occurred on the brace for T-joint specimens, with β ranging from 0.75 to 1.00. Table 7 shows that the peak SCF of each specimen reduced due to the concrete infill of the chord. The experimental reduction percentages in peak SCFs due to concrete infill for S6S1, S5S1, S6S2, S6S3, S6S4, S6S5, and S5S5 are 11.48, 10.77, 28.44, 37.15, 20.62, 11.48, and 35.16, respectively. The overall mean of the experimental reduction percentage in peak SCF due to concrete infill is 22.16%.

The SCFs determined from the FEA of the seven concrete-filled T-joint models under in-plane bending are shown in Table 8. These values were compared with the empty T-joint models reported by [7]. The peak numerical SCFs of the concrete-filled SHS T-joints occurred on the chord, with β ranging from 0.25 to 0.50. The peak numerical SCF of the concrete-filled T-joints occurred on the brace member, with β ranging from 0.75 to 1.00. There are different locations of maximum SCF due to the variation of the non-dimensional parameters 2γ and τ which relate to the differences in slenderness and rigidity between specimens. The numerical results showed that the peak SCF of each specimen reduced due to the concrete infill of the chord. The numerical reduction percentage in peak SCFs ranges from 4.26 to 43.64. The overall mean of the numerical reduction percentage in peak SCF due to concrete infill is 18.89%.

Table 9 shows that there is a good agreement between the peak SCF in the FEA model and that obtained experimentally. The ratios of peak SCFs in the FEA model to that in the experiment for S6S1, S5S1, S6S2, and S6S4 are 0.94, 0.60, 1.19, and 0.87, respectively. The ratios of the peak SCF in FEA model to that in the experiment for S6S3, S6S5, and S5S5 are 1.37, 1.24, and 1.80, respectively. These ratios show that the peak numerical SCF is higher than the corresponding experimental SCFs. As the maximum numerical SCFs for S6S3, S6S5, and S5S5 are higher than the corresponding experimental SCFs, a safer design formula for predicting the maximum SCFs was proposed. As shown in Table 9, the peak experimental and corresponding numerical SCFs generally occurred in the same location.

After validating the FE models, a parametric study was conducted on SCFs of concrete-filled SHS-SHS T-joints under in-plane bending using Abaqus FEA software. The aim of the parametric study was to evaluate the effects of the non-dimensional parameters on the SCFs. The SCFs at the hot spot locations of the 60 concrete-filled SHS-SHS T-joints determined from the FEA are shown in Table 10. As shown in Table 10, maximum SCFs of the concrete-filled T-joints generally occurred along line C. Experimental investigation on 7 cold-formed SHS T-joints with concrete-filled chords under in-plane bending loads was conducted to determine SCFs and validate the FEA results. However, an experimental investigation is a long process, costly, and often has human errors. Finite element modeling of concrete-filled SHS T-joints using Abaqus FEA software provides a low-cost solution within a relatively short time and prevents human error in the experimental lab. Researchers and engineers can easily determine the life of welded composite tubular structures with concrete-filled chord T-joints using the design graphs and formulae proposed in this paper. This will save time and cost as experimental and numerical investigations will not be required to evaluate the life of welded composite tubular structures. Fatigue tests on SHS-SHS T-joints with concrete-filled chords under axial loading, in-plane bending, and out-of-plane bending on the brace will be recommended for future research studies.

### 4.2. Influence of β on SCFs

**Figure 8:**
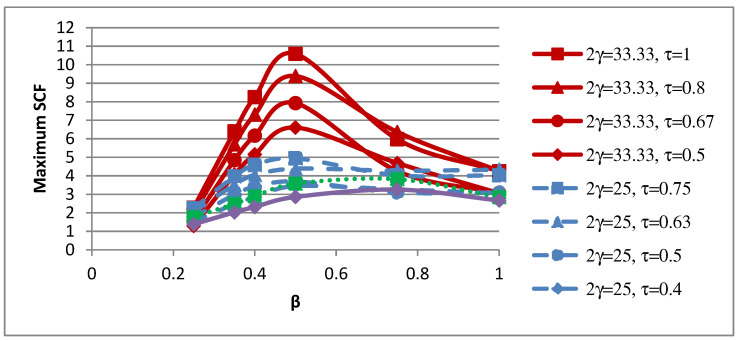
Influence of β on peak SCFs.

**Figure 9:**
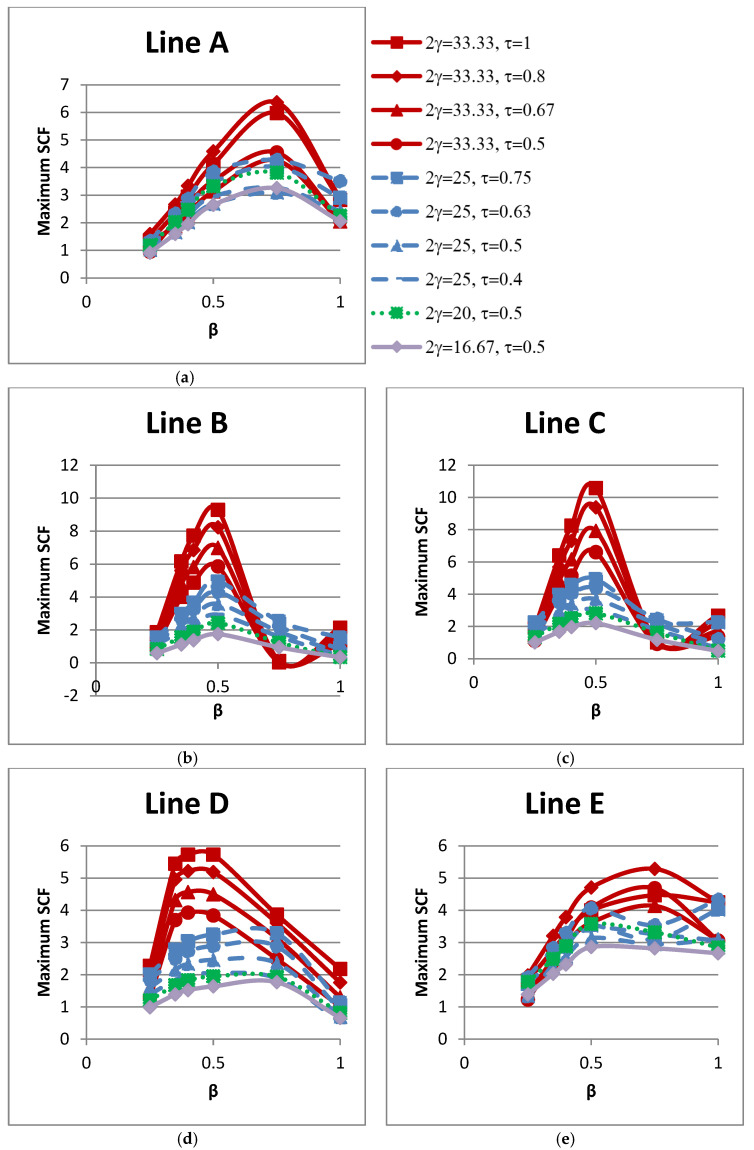
Influence of β on SCFs along lines: (**a**) A; (**b**) B; (**c**) C; (**d**) D; and (**e**) E.

The influence of the non-dimensional parameter β on peak SCFs of concrete-filled SHS-SHS T-joints under in-plane brace bending is shown in Figure 8 and Table 11. The peak SCFs in concrete-filled SHS T-joints (with 2γ = 25, 33) occurred when β = 0.5. For T-joints with 2γ = 25 and 33, the maximum SCFs increased with the increase in value of β and decreased when β = 0.75. The peak SCFs in concrete-filled SHS T-joints (with 2γ = 20, 16.67) occurred when β = 0.75. Furthermore, Figure 9 shows the influence of β on SCFs along lines A–E. Figure 9 shows that SCF trends along lines A and E located on the brace are similar and non-linear. In general, the SCFs trends along lines B, C, and D, which are located on the chord, are comparable.

### 4.3. Influence of τ on SCFs

**Figure 10:**
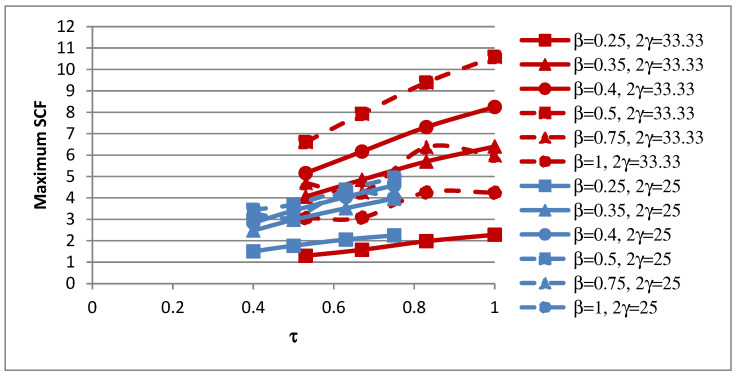
Influence of τ on peak SCFs.

**Figure 11:**
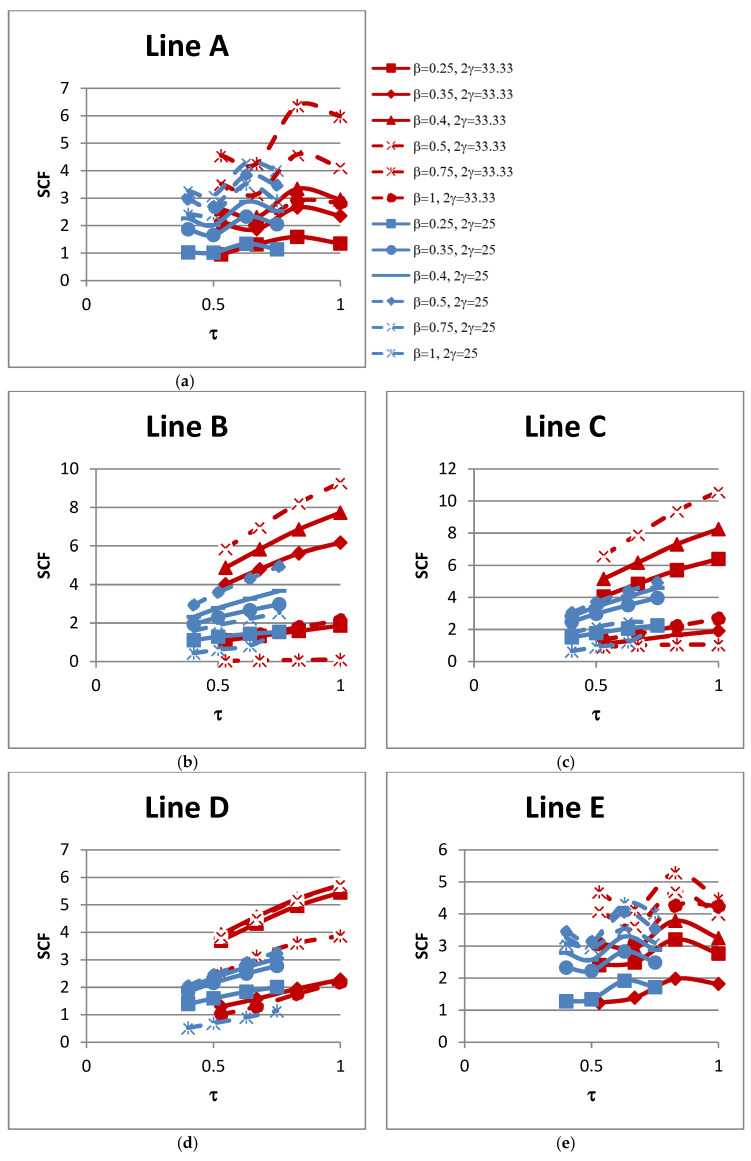
Influence of τ on SCFs along lines: (**a**) A; (**b**) B; (**c**) C; (**d**) D; and (**e**) E.

The influence of the non-dimensional parameter τ on the peak SCFs of concrete-filled SHS-SHS T-joint connections is shown in Figure 10 and Table 12. The peak SCFs generally increased with the increase in value of τ. The influence of τ on the SCFs along lines A–E is shown in Figure 11. The trends of the SCFs along lines A and E, which are located on the brace, are similar and both are non-linear. In general, the SCFs on the chord along lines B, C, and D generally increased linearly with the increase in value of τ.

### 4.4. Influence of 2γ on SCFs

**Figure 12:**
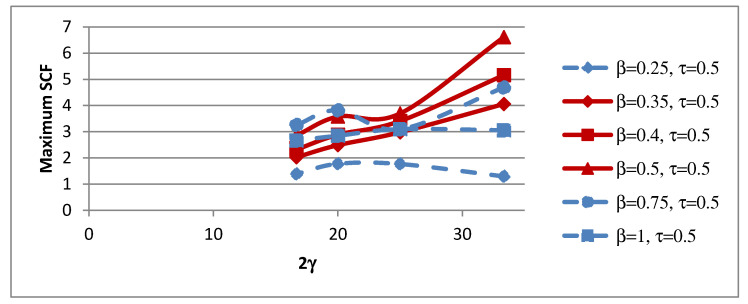
Influence of 2γ on maximum SCFs.

**Figure 13:**
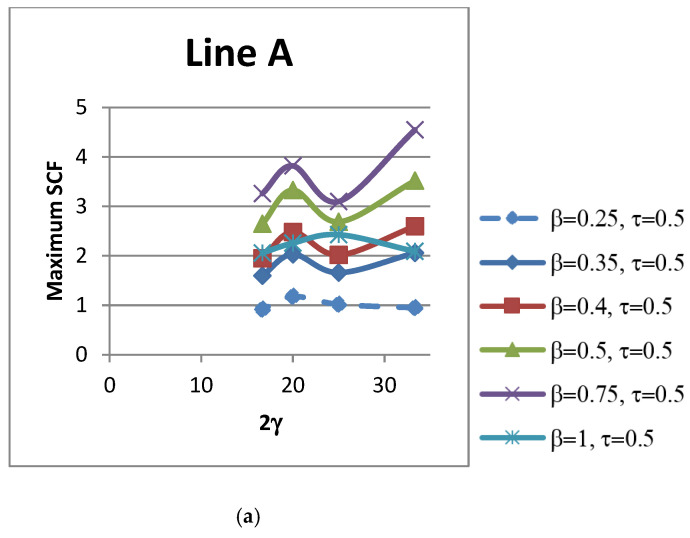

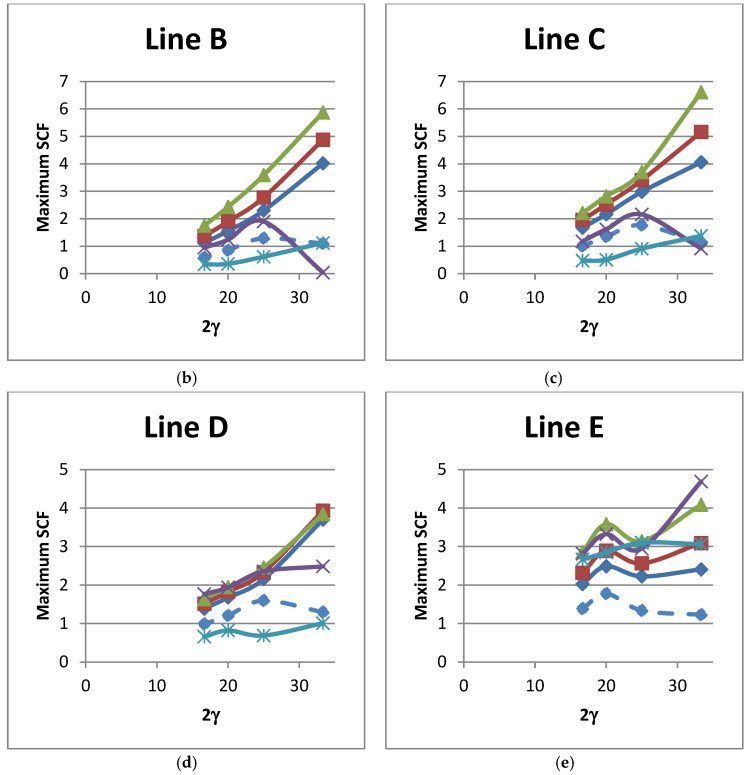
Influence of 2γ on SCFs along lines: (**a**) A; (**b**) B; (**c**) C; (**d**) D; and (**e**) E.

The non-dimensional parameter 2γ is defined as the ratio of the chord width to chord wall thickness (b_0_/t_0_). The influence of the non-dimensional parameter 2γ on peak SCFs of concrete-filled SHS-SHS T-joints is shown in Figure 12 and Table 13. The influence of 2γ on SCFs along lines A–E under in-plane brace bending is shown in Figure 13. The trends of the SCFs are both linear and non-linear.

## 5. Proposed Design Equations

Design equations for predicting the SCFs of SHS-SHS T-joints with concrete-filled chords under in-plane bending on the brace are proposed. The design equations for SCFs at lines A–E were proposed based on Equation (3) by CIDECT Design Guide 8 [6]. Equation (3) is based on the work of [23]. Non-linear multiple regression analysis was carried out to determine the values of the constants *a–h*. Table 14 shows the values of the constants *a–h* for lines A–E and for peak SCFs. Using Equation (4), the predicted peak SCF of concrete-filled SHS T-joints under in-plane bending were calculated; see Table 15. There is a good agreement between the peak SCFs determined from the predicted formula and FEA. As shown in Table 15, the coefficient of variation of the overall ratio is 0.17.

(3) $${\mathrm{SCF}}_{\mathrm{SHS}}=\left(a+b\beta +c\beta^{2}+d2\gamma \right){\left(2\gamma \right)}^{\left(e+f\beta +g\beta^{2}\right)}\tau^{h}$$

(4) $${\mathrm{SCF}}_{\mathrm{Peak}}=\left(0.23326-0.46734\beta +0.25852\beta^{2}-0.00060\left(2\gamma \right)\right){\left(2\gamma \right)}^{\left(0.09625+3.97770\beta -2.10467\beta^{2}\right)}\tau^{0.70022}$$

(5) $${\mathrm{SCF}}_{A}=\left(5.68385-11.16130\beta +5.66213\beta^{2}-0.00410\left(2\gamma \right)\right){\left(2\gamma \right)}^{\left(-0.85699+2.76055\beta -0.73792{g\beta }^{2}\right)}\tau^{0.45556}$$

(6) $${\mathrm{SCF}}_{B}=\left(0.00667-0.01483\beta +0.00997\beta^{2}-0.00003\left(2\gamma \right)\right){\left(2\gamma \right)}^{\left(0.46519+7.32706\beta -5.50964\beta^{2}\right)}\tau^{0.73617}$$

(7) $${\mathrm{SCF}}_{C}=\left(0.06907-0.15705\beta +0.10148\beta^{2}-0.00023\left(2\gamma \right)\right){\left(2\gamma \right)}^{\left(-0.05171+6.57518\beta -4.79370\beta^{2}\right)}\tau^{0.75048}$$

(8) $${\mathrm{SCF}}_{D}=\left(0.06796-0.17959\beta +0.13975\beta^{2}-0.00023\left(2\gamma \right)\right){\left(2\gamma \right)}^{\left(0.02716+6.55388\beta -5.28517\beta^{2}\right)}\tau^{0.68201}$$

(9) $${\mathrm{SCF}}_{E}=\left(4.58425-9.54630\beta +5.07597\beta^{2}-0.00228\left(2\gamma \right)\right){\left(2\gamma \right)}^{\left(-0.63085+2.29810\beta -0.33863\beta^{2}\right)}\tau^{0.24647}$$

**Figure 14:**
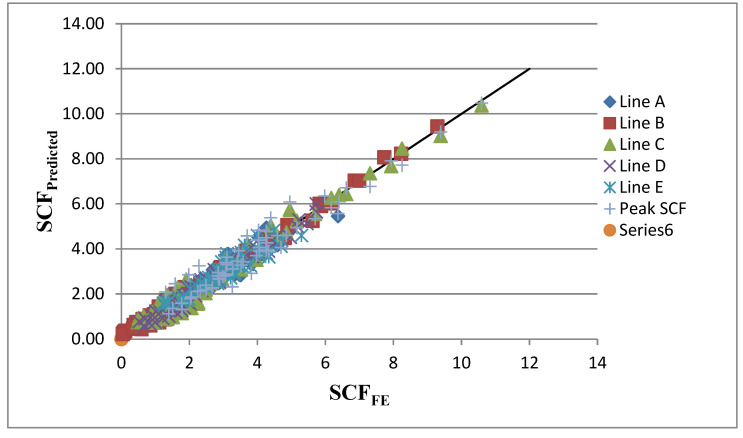
Comparison of SCFs from the predicted design equations and FEA.

Based on Equations (5)–(9), the predicted SCFs at lines A–E of SHS-SHS T-joints with concrete-filled chords under in-plane bending are shown in Table 16. From Table 16 and Figure 14, it can be observed that there is a good agreement between the SCFs determined from the predicted design equations and the FEA. Some high ratios of the predicted SCF to that in FE can be observed along line B, as the predicted SCF and corresponding FE SCF are relatively small (less than 1). As shown in Table 16, the acceptable coefficient of variation (COV) of predicted SCF to FE SCF for each line is observed.

**Figure 15:**
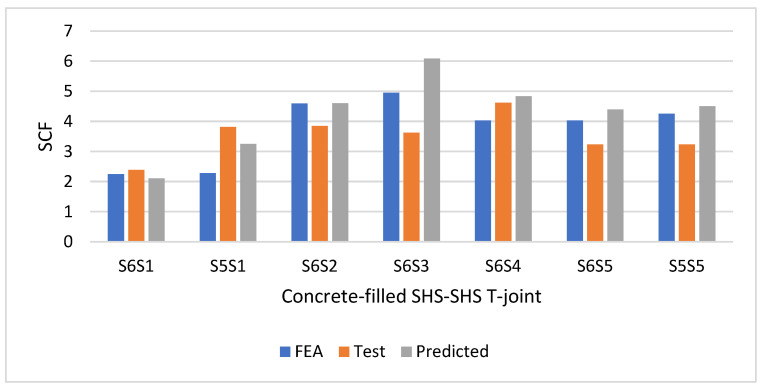
Comparison of SCFs from the FEA, test, and predicted design equations.

Figure 15 shows the SCFs of concrete-filled SHS-SHS T-joint specimens determined from FEA, test, and predicted design equations. Comparable SCF results were obtained between the numerical SCFs, with SCFs calculated from the test and proposed design equations.

## 6. Conclusions

In total, seven cold-formed SHS-SHS T-joints with concrete-filled chords were tested experimentally using strain gauges and then numerically determined using Abaqus FEA software. The experimental and numerical SCFs were determined at the hot spot locations (lines A–E) under static in-plane brace bending. After validating the FEA results, a parametric study was conducted on 60 concrete-filled welded tubular SHS-SHS T-joints to study the effects of the non-dimensional parameters on the SCFs. The non-dimensional parameters of the 60 concrete-filled SHS-SHS T-joints are as follows: 0.25≤β≤1; 16.67≤2γ≤33.3; and 0.4≤τ≤1. These non-dimensional parameters extend the non-dimensional parameters previously reported by [1]. The T-joints models were subjected to static in-plane bending on the brace. The distribution of the SCFs at the hot spot locations (lines A–E) was determined and the effect of the non-dimensional parameters (β, 2γ and τ) on the SCFs was discussed. Design formulae for predicting the SCF at the hot spot locations of concrete-filled SHS-SHS T-joints under in-plane brace bending were proposed. Based on the work carried out in this research, the following conclusion was made, there is good agreement between the experimental nominal stresses and the nominal stresses from the simple beam theory are observed. The numerical nominal stresses agree with the values determined from the simple beam theory. The peak experimental and FE SCFs of the concrete-filled SHS T-joints occurred on the chord, with β ranging from 0.25 to 0.50. The peak experimental and FE SCFs occurred on the brace for T-joints, with β ranging from 0.75 to 1.00.

The SCF of the 7 T-joint specimens with concrete-filled chords were compared with the empty T-joints reported by [7]. The peak experimental SCFs of the empty and concrete-filled SHS T-joints occurred on the chord, with β ranging from 0.25 to 0.50. The peak SCF occurred on the brace for T-joints, with β ranging from 0.75 to 1.00. The peak SCF of each specimen was reduced due to the concrete infill of the chord. The experimental reduction percentage in peak SCFs ranged from 10.77% to 37.15%. The overall mean of the experimental reduction percentage in peak SCF due to concrete infill was 22.16%.

The SCFs determined from the Abaqus FEA software of the 7 concrete-filled T-joint models under in-plane bending were compared with the empty T-joint models reported by [7]. The peak numerical SCFs of the concrete-filled SHS T-joints occurred on the chord, with β ranging from 0.25 to 0.50. The peak numerical SCFs of the concrete-filled T-joints occurred on the brace member, with β ranging from 0.75 to 1.00. The FE results showed that the peak SCF of each specimen was reduced due to the concrete infill of the chord. The numerical reduction percentage in peak SCFs ranged from 4.26% to 43.64%. The overall mean of the numerical reduction percentage in peak SCF due to concrete infill was 18.89%. These observations are similar to the experimental results. Good agreement is observed between the FE SCFs with SCFs calculated from the test.

The non-dimensional parameters have a significant effect on SCFs. The maximum SCFs in concrete-filled SHS-SHS T-joints under in-plane bending occurred when β = 0.5 and 0.75. The peak SCFs generally increased with the increase value of τ. This paper proposed design graphs and design formulae for predicting the SCF of concrete-filled SHS-SHS T-joints subject to in-plane brace bending. Comparable results were obtained between the FE SCFs with SCFs calculated from the proposed design equations.

The results of this research will contribute to the fatigue design and performance evaluation of existing and new tubular structures with concrete-filled SHS-SHS T-joints. This paper will allow researchers and engineers to determine the life of welded composite tubular structures using the proposed design formulae.

## Figures and Tables

**Table 1:** SHS T-joint specimens used in the experimental investigation.

Series	Chord	Brace	Non-Dimensional Parameters		
	$d_{o}\;\times \;b_{o}\;\times \;t_{o}$ (mm × mm × mm)	$d_{1}\;\times \;b_{1}\;\times \;t_{1}$ (mm × mm × mm)	$\beta =\frac{b_{1}}{b_{o}}$	$2\gamma =\frac{b_{o}}{t_{o}}$	$\tau =\frac{t_{1}}{t_{o}}$
S6S1	100 × 100 × 4 SHS	25 × 25 × 3 SHS	0.25	25.00	0.75
S5S1	100 × 100 × 3 SHS	25 × 25 × 3 SHS	0.25	33.33	1.00
S6S2	100 × 100 × 4 SHS	40 × 40 × 3 SHS	0.40	25.00	0.75
S6S3	100 × 100 × 4 SHS	50 × 50 × 3 SHS	0.50	25.00	0.75
S6S4	100 × 100 × 4 SHS	75 × 75 × 3 SHS	0.75	25.00	0.75
S6S5	100 × 100 × 4 SHS	100 × 100 × 3 SHS	1.00	25.00	0.75
S5S5	100 × 100 × 3 SHS	100 × 100 × 3 SHS	1.00	33.33	1.00

**Table 2:** Experimental nominal strains of specimen S6S4 under in-plane bending.

Extrapolation Points from the Load’s Location (mm)	In-Plane Bending Load (kN)			
	0.50	0.71	0.92	1.21
	Strain (µε)			
575	0	0	0	0
375	21.35	31.85	41.28	53.08
250	37.60	54.89	70.60	92.09
125	58.41	83.17	105.34	135.96
**Nominal strain**	71.83	103.11	131.24	169.84

**Table 3:** Nominal stresses from experiment and simple beam theory.

Test Series	Load, F (N)	Height, H (mm)	Elastic Section Modulus, Z (mm^3^)	Nominal Strain, (µε)	Young’s Modulus, E (MPa)	σ_nom.exp_ = Eε, (MPa)	$\sigma_{\mathrm{nom}.\mathrm{BT}}\;=\;\frac{F\times H}{Z},$ (MPa)	Ratio $\frac{\sigma_{nom.exp}}{\sigma_{nom.BT}}$
S6S1	193.003	575	1470	325.05	222,576.5	72.35	75.49	0.96
	214.451	575	1470	358.85	222,576.5	79.87	83.88	0.95
	238.82	575	1470	394.34	222,576.5	87.77	93.42	0.94
	256.133	575	1470	420.40	222,576.5	93.57	100.19	0.93
S5S1	98.647	575	1470	168.61	189,604.0	31.97	38.59	0.83
	109.655	575	1470	187.59	189,604.0	35.57	42.89	0.83
	129.065	575	1470	224.31	189,604.0	42.53	50.48	0.84
	139.687	575	1470	265.59	189,604.0	50.36	54.64	0.92
S6S2	150.842	575	4660	85.02	214,680.5	18.25	18.61	0.98
	195.461	575	4660	109.95	214,680.5	23.60	24.12	0.98
	295.729	575	4660	163.90	214,680.5	35.19	36.49	0.96
	397.128	575	4660	219.79	214,680.5	47.18	49.00	0.96
S6S3	192.021	575	7790	63.349	215,207.0	13.63	14.17	0.96
	298.084	575	7790	97.66	215,207.0	21.02	22.00	0.96
	394.009	575	7790	129.23	215,207.0	27.81	29.08	0.96
	494.863	575	7790	160.4	215,207.0	34.52	36.53	0.95
S6S4	495.543	575	19,100	71.83	205,234.0	14.74	14.92	0.99
	714.112	575	19,100	103.11	205,234.0	21.16	21.50	0.98
	915.075	575	19,100	131.24	205,234.0	26.93	27.55	0.98
	1208.042	575	19,100	169.84	205,234.0	34.86	36.37	0.96
S6S5	847.142	575	35,400	70.80	207,907.0	14.72	13.76	1.07
	2040.564	575	35,400	157.98	207,907.0	32.85	33.14	0.99
	3140.656	575	35,400	245.20	207,907.0	50.98	51.01	1.00
	3585.205	575	35,400	281.57	207,907.0	58.54	58.23	1.01
S5S5	1021.493	575	35,400	81.43	207,907.0	16.93	16.59	1.02
	1473.567	575	35,400	114.38	207,907.0	23.78	23.94	0.99
	2004.407	575	35,400	152.98	207,907.0	31.81	32.56	0.98
	2500.394	575	35,400	190.08	207,907.0	39.52	40.61	0.97
Average								0.96
COV								0.06

Note: σ_nom.exp_ = experimental nominal stress; and σ_nom.BT_ = simple beam theory nominal stress.

**Table 4:** Experimental SCFs of concrete-filled SHS-SHS T-joints under in-plane brace bending.

Series	Hot Spot Locations	Load (kN)	HSSN (µε)	Nominal Strain (µε)	SNCF	SNCF_SHS_	SCF_Test_
S6S1	Line A	0.19	181.29	325.05	0.56	0.60	0.66
		0.21	210.59	358.85	0.59		
		0.24	243.77	394.34	0.62		
		0.26	265.62	420.40	0.63		
	Line B	0.19	360.72	325.05	1.11	1.17	1.29
		0.21	413.09	358.85	1.15		
		0.24	472.59	394.34	1.20		
		0.26	515.54	420.40	1.23		
	Line C	0.19	633.29	325.05	1.95	1.97	2.17
		0.21	703.03	358.85	1.96		
		0.24	781.15	394.34	1.98		
		0.26	835.23	420.40	1.99		
	Line D	0.19	717.1	325.05	2.21	2.18	2.39
		0.21	782.54	358.85	2.18		
		0.24	853.59	394.34	2.16		
		0.26	903.73	420.40	2.15		
	Line E	0.19	596.90	325.05	1.84	1.82	2.00
		0.21	652.27	358.85	1.82		
		0.24	713.26	394.34	1.81		
		0.26	756.48	420.40	1.80		
S5S1	Line A	0.10	147.89	168.61	0.88	0.88	0.97
		0.11	166.99	187.59	0.89		
		0.13	205.93	224.31	0.92		
		0.14	226.10	265.59	0.85		
	Line B	0.10	197.44	168.61	1.17	1.23	1.35
		0.11	229.15	187.59	1.22		
		0.13	289.46	224.31	1.29		
		0.14	324.06	265.59	1.22		
	Line C	0.10	579.74	168.61	3.44	3.46	3.81
		0.11	657.33	187.59	3.50		
		0.13	803.35	224.31	3.58		
		0.14	881.63	265.59	3.32		
	Line D	0.10	542.85	168.61	3.22	3.09	3.40
		0.11	598.07	187.59	3.19		
		0.13	701.28	224.31	3.13		
		0.14	753.93	265.59	2.84		
	Line E	0.10	218.30	168.61	1.29	1.24	1.36
		0.11	239.16	187.59	1.27		
		0.13	280.99	224.31	1.25		
		0.14	301.75	265.59	1.14		
S6S2	Line A	0.15	236.00	85.02	2.78	2.82	3.11
		0.20	305.72	109.95	2.78		
		0.30	466.53	163.90	2.85		
		0.40	635.96	219.79	2.89		
	Line B	0.15	288.42	85.02	3.39	3.50	3.85
		0.20	376.60	109.95	3.43		
		0.30	582.69	163.90	3.56		
		0.40	794.84	219.79	3.62		
	Line C	0.15	286.38	85.02	3.37	3.34	3.67
		0.20	366.93	109.95	3.34		
		0.30	547.33	163.90	3.34		
		0.40	726.63	219.79	3.31		
	Line D	0.15	235.79	85.02	2.77	2.70	2.96
		0.20	299.44	109.95	2.72		
		0.30	437.11	163.90	2.67		
		0.40	575.04	219.79	2.62		
	Line E	0.15	259.57	85.02	3.05	3.02	3.32
		0.20	332.01	109.95	3.02		
		0.30	493.74	163.90	3.01		
		0.40	657.13	219.79	2.99		
S6S3	Line A	0.19	143.20	63.35	2.26	2.75	3.02
		0.30	265.00	97.66	2.71		
		0.39	378.47	129.23	2.93		
		0.49	495.32	160.40	3.09		
	Line B	0.19	135.02	63.35	2.13	2.75	3.03
		0.30	263.59	97.66	2.70		
		0.39	384.87	129.23	2.98		
		0.49	514.40	160.40	3.21		
	Line C	0.19	181.21	63.35	2.86	3.29	3.62
		0.30	321.17	97.66	3.29		
		0.39	444.86	129.23	3.44		
		0.49	574.99	160.40	3.58		
	Line D	0.19	146.27	63.35	2.31	2.40	2.64
		0.30	235.30	97.66	2.41		
		0.39	313.26	129.23	2.42		
		0.49	394.08	160.40	2.46		
	Line E	0.19	194.77	63.35	3.07	2.98	3.28
		0.30	292.58	97.66	3.00		
		0.39	379.70	129.23	2.94		
		0.49	469.41	160.40	2.93		
S6S4	Line A	0.50	305.66	71.83	4.26	4.20	4.62
		0.71	433.90	103.11	4.21		
		0.92	546.25	131.24	4.16		
		1.21	707.35	169.84	4.16		
	Line B	0.50	114.60	71.83	1.60	1.64	1.80
		0.71	166.95	103.11	1.62		
		0.92	216.70	131.24	1.65		
		1.21	286.61	169.84	1.69		
	Line C	0.50	312.58	71.83	4.35	4.15	4.57
		0.71	432.08	103.11	4.19		
		0.92	535.78	131.24	4.08		
		1.21	675.22	169.84	3.98		
	Line D	0.50	123.56	71.83	1.72	1.64	1.80
		0.71	170.94	103.11	1.66		
		0.92	210.24	131.24	1.60		
		1.21	266.28	169.84	1.57		
	Line E	0.50	160.33	71.83	2.23	2.21	2.43
		0.71	226.15	103.11	2.19		
		0.92	287.16	131.24	2.19		
		1.21	376.02	169.84	2.21		
S6S5	Line A	0.85	160.91	70.80	2.27	2.00	2.20
		2.04	315.98	157.98	2.00		
		3.14	462.98	245.20	1.89		
		3.59	522.55	281.57	1.86		
	Line B	0.85	49.08	70.80	0.69	0.47	0.52
		2.04	72.23	157.98	0.46		
		3.14	92.48	245.20	0.38		
		3.59	103.11	281.57	0.37		
	Line C	0.85	67.69	70.80	0.96	0.79	0.86
		2.04	123.80	157.98	0.78		
		3.14	174.83	245.20	0.71		
		3.59	193.81	281.57	0.69		
	Line D	0.85	59.96	70.80	0.85	0.76	0.84
		2.04	118.29	157.98	0.75		
		3.14	179.67	245.20	0.73		
		3.59	203.50	281.57	0.72		
	Line E	0.85	209.33	70.80	2.96	2.95	3.24
		2.04	468.46	157.98	2.97		
		3.14	719.99	245.20	2.94		
		3.59	824.00	281.57	2.93		
S5S5	Line A	1.02	151.38	81.43	1.86	1.96	2.16
		1.47	224.19	114.38	1.96		
		2.00	308.05	152.98	2.01		
		2.50	385.17	190.08	2.03		
	Line B	1.02	78.22	81.43	0.96	0.87	0.96
		1.47	101.00	114.38	0.88		
		2.00	128.69	152.98	0.84		
		2.50	151.66	190.08	0.80		
	Line C	1.02	58.21	81.43	0.71	0.61	0.67
		1.47	75.85	114.38	0.66		
		2.00	88.31	152.98	0.58		
		2.50	93.03	190.08	0.49		
	Line D	1.02	38.11	81.43	0.47	0.51	0.56
		1.47	58.52	114.38	0.51		
		2.00	80.84	152.98	0.53		
		2.50	101.63	190.08	0.53		
	Line E	1.02	160.07	81.43	1.97	2.14	2.36
		1.47	238.58	114.38	2.09		
		2.00	329.62	152.98	2.15		
		2.50	448.44	190.08	2.36		

**Table 5:** Numerical and simple beam theory nominal stresses.

Series Name	D_B.MID_ _(mm)_	σ_B.MID_ _(MPa)_	D_B.QTR_ _(mm)_	σ_B.QTR_ _(MPa)_	D_B.END_ _(mm)_	σ_nom.FEA_ Numerical (FEA) _(MPa)_	σ_nom.BT_ Beam Theory _(MPa)_	Ratio $\frac{\sigma_{nom.BT}}{\sigma_{nom.num}}$
S6S1	250	99.39	375	51.27	0	195.62	208.16	1.06
S5S1	250	99.39	375	51.27	0	195.62	208.16	1.06
S6S2	250	32.10	375	16.56	0	63.19	65.67	1.04
S6S3	250	19.36	375	9.99	0	38.11	39.28	1.03
S6S4	250	7.97	375	4.11	0	15.70	16.02	1.02
S6S5	250	4.32	375	2.23	0	8.51	8.64	1.02
S5S5	250	4.32	375	2.23	0	8.51	8.64	1.02
Average								1.04
COV								0.02

Note: D_B.MID_ = distance brace middle; σ_B.MID_ = stress brace middle; D_B.QTR_ = distance brace quarter; σ_B.QTR_ = stress brace quarter; D_B.END_ = distance brace end; σ_nom.BT_ = simple beam theory nominal stress; and σ_nom.FEA_ = numerical nominal stress.

**Table 6:** Numerical SCFs of SHS-SHS T-joint specimens under out-of-plane bending.

Series	Hot Spot Locations	Load (kN)	Hot Spot Stress (MPa)	Nominal Stress (MPa)	SCF_FEA_
S6S1	Line A	0.6	223.14	195.62	1.14
	Line B		300.91	195.62	1.54
	Line C		440.38	195.62	2.25
	Line D		393.94	195.62	2.01
	Line E		336.25	195.62	1.72
S5S1	Line A	0.6	264.40	195.62	1.35
	Line B		365.44	195.62	1.87
	Line C		372.70	195.62	1.91
	Line D		445.60	195.62	2.28
	Line E		356.02	195.62	1.82
S6S2	Line A	0.6	160.18	63.19	2.53
	Line B		232.19	63.19	3.67
	Line C		290.24	63.19	4.59
	Line D		192.33	63.19	3.04
	Line E		181.97	63.19	2.88
S6S3	Line A	0.6	132.17	38.11	3.47
	Line B		188.55	38.11	4.95
	Line C		188.74	38.11	4.95
	Line D		123.95	38.11	3.25
	Line E		134.57	38.11	3.53
S6S4	Line A	0.6	63.24	15.69	4.03
	Line B		39.85	15.69	2.54
	Line C		37.88	15.69	2.41
	Line D		51.63	15.69	3.29
	Line E		48.61	15.69	3.10
S6S5	Line A	0.6	24.71	8.51	2.91
	Line B		13.13	8.51	1.54
	Line C		19.26	8.51	2.26
	Line D		9.71	8.51	1.14
	Line E		34.30	8.51	4.03
S5S5	Line A	0.6	24.10	8.51	2.83
	Line B		18.23	8.51	2.14
	Line C		22.71	8.51	2.67
	Line D		18.53	8.51	2.18
	Line E		36.12	8.51	4.25

**Table 7:** Experimental SCFs of concrete-filled T-joints under in-plane bending.

Series Name	Non-Dimensional Parameters			Empty/Concrete	Experimental SCF (Quadratic)					Peak SCF	Reduction % in Peak SCF
	β	2γ	τ		Chord			Brace			
					B	C	D	A	E		
S6S1	0.25	25	0.75	Empty	**2.07**	2.58	**2.70**	0.28	2.22	**2.70**	11.48
				Concrete	1.29	2.17	**2.39**	0.66	2.00	**2.39**	
S5S1	0.25	33.33	1.00	Empty	1.37	**4.27**	3.55	0.90	2.37	**4.27**	10.77
				Concrete	1.35	**3.81**	3.40	0.97	1.36	**3.81**	
S6S2	0.40	25	0.75	Empty	3.38	4.65	**5.38**	2.41	3.06	**5.38**	28.44
				Concrete	**3.85**	3.67	2.96	3.11	3.32	**3.85**	
S6S3	0.50	25	0.75	Empty	4.45	5.04	**5.76**	3.57	3.54	**5.76**	37.15
				Concrete	3.03	**3.62**	2.64	3.02	3.28	**3.62**	
S6S4	0.75	25	0.75	Empty	1.19	4.90	2.80	**5.82**	3.07	**5.82**	20.62
				Concrete	1.80	4.57	1.80	**4.62**	2.43	**4.62**	
S6S5	1.00	25	0.75	Empty	1.12	1.25	1.16	2.34	**3.66**	**3.66**	11.48
				Concrete	0.52	0.86	0.84	2.20	**3.24**	**3.24**	
S5S5	1.00	33.33	1.00	Empty	3.08	2.29	1.49	**3.64**	2.90	**3.64**	35.16
				Concrete	0.96	0.67	0.56	2.16	**2.36**	**2.36**	
Mean											22.16

**Table 8:** Numerical SCFs of concrete-filled T-joints under in-plane bending.

Series Name	Stress Concentration Factor (SCF)										Reduction % in Peak SCF
	Empty SHS T-Joints Matti and Mashiri [7]					Concrete-Filled SHS T-Joints					
	B	C	D	A	E	B	C	D	A	E	
S6S1	1.16	**2.35**	2.28	0.92	1.82	1.54	**2.25**	2.01	1.14	1.72	4.26
S5S1	2.18	2.50	**3.11**	1.03	1.98	1.87	1.91	**2.28**	1.35	1.82	26.69
S6S2	3.34	**5.43**	4.02	2.37	3.17	3.67	**4.59**	3.04	2.53	2.88	15.47
S6S3	5.00	**6.02**	4.55	3.61	4.03	4.95	**4.95**	3.25	3.47	3.53	17.77
S6S4	**7.15**	5.06	4.92	5.24	3.89	2.54	2.41	3.29	**4.03**	3.10	43.64
S6S5	1.55	2.31	1.23	3.82	**4.58**	1.54	2.26	1.14	2.91	**4.03**	12.01
S5S5	2.30	3.24	2.19	4.29	**4.85**	2.14	2.67	2.18	2.83	**4.25**	12.37
Average											18.89

**Table 9:** Experimental and numerical SCFs of concrete-filled T-joints under in-plane bending.

Series Name	Non-Dimensional Parameters			FEA/ Test	SCF (Quadratic)					Maximum SCF	Ratio of Maximum SCFs
	$\beta \;=\;\frac{b_{1}}{b_{o}}$	$2\gamma \;=\;\frac{b_{o}}{t_{o}}$	$\tau \;=\;\frac{t_{1}}{t_{o}}$		Chord			Brace			$\frac{SCF_{ABAQUS}}{SCF_{Experiment}}$
					B	C	D	A	E		
S6S1	0.25	25	0.75	FEA	1.54	**2.25**	2.01	1.14	1.72	2.25	0.94
				Test	1.29	2.17	**2.39**	0.66	2.00	2.39	
S5S1	0.25	33.33	1.00	FEA	1.87	1.91	**2.28**	1.35	1.82	2.28	0.60
				Test	1.35	**3.81**	3.40	0.97	1.36	3.81	
S6S2	0.40	25	0.75	FEA	3.67	**4.59**	3.04	2.53	2.88	4.59	1.19
				Test	**3.85**	3.67	2.96	3.11	3.32	3.85	
S6S3	0.50	25	0.75	FEA	**4.95**	**4.95**	3.25	3.47	3.53	4.95	1.37
				Test	3.03	**3.62**	2.64	3.02	3.28	3.62	
S6S4	0.75	25	0.75	FEA	2.54	2.41	3.29	**4.03**	3.10	4.03	0.87
				Test	1.80	4.57	1.80	**4.62**	2.43	4.62	
S6S5	1.00	25	0.75	FEA	1.54	2.26	1.14	2.91	**4.03**	4.03	1.24
				Test	0.52	0.86	0.84	2.20	**3.24**	3.24	
S5S5	1.00	33.33	1.00	FEA	2.14	2.67	2.18	2.83	**4.25**	4.25	1.80
				Test	0.96	0.67	0.56	2.16	**2.36**	2.36	
Average											1.15

**Table 10:** SCFs obtained from FEA.

Series Name	SHS Chord	SHS Brace	Non-Dimensional Parameters			SCF_CFSHS.FE_				
	$d_{o}\;\times \;b_{o}\;\times \;t_{o}$	$d_{1}\;\times \;b_{1}\;\times \;t_{1}$								
	mm × mm × mm	mm × mm × mm	β	2γ	τ	A	B	C	D	E
S21S1	100×100×3 SHS	25×25×3 SHS	0.25	33.33	1.0	1.35	1.87	1.91	**2.28**	1.82
S21S2	100×100×3 SHS	25×25×2.5 SHS	0.25	33.33	0.83	1.59	1.59	1.65	1.95	**1.98**
S21S3	100×100×3 SHS	25×25×2 SHS	0.25	33.33	0.67	1.32	1.33	1.35	**1.58**	1.39
S21S4	100×100×3 SHS	25×25×1.6 SHS	0.25	33.33	0.53	0.95	1.10	1.11	**1.30**	1.23
S21S5	100×100×3 SHS	35×35×3 SHS	0.35	33.33	1.0	2.36	6.17	**6.40**	5.45	2.77
S21S6	100×100×3 SHS	35×35×2.5 SHS	0.35	33.33	0.83	2.66	5.62	**5.70**	4.96	3.21
S21S7	100×100×3 SHS	35×35×2 SHS	0.35	33.33	0.67	1.87	4.80	**4.85**	4.32	2.49
S21S8	100×100×3 SHS	35×35×1.6 SHS	0.35	33.33	0.53	2.06	4.02	**4.06**	3.70	2.41
S21S9	100×100×3 SHS	40×40×3 SHS	0.4	33.33	1.0	2.95	7.73	**8.25**	5.73	3.25
S21S10	100×100×3 SHS	40×40×2.5 SHS	0.4	33.33	0.83	3.34	6.86	**7.31**	5.22	3.79
S21S11	100×100×3 SHS	40×40×2 SHS	0.4	33.33	0.67	2.31	5.83	**6.17**	4.57	2.94
S21S12	100×100×3 SHS	40×40×1.6 SHS	0.4	33.33	0.53	2.60	4.88	**5.16**	3.93	3.09
S21S13	100×100×3 SHS	50×50×3 SHS	0.5	33.33	1.0	4.12	9.29	**10.59**	5.73	4.00
S21S14	100×100×3 SHS	50×50×2.5 SHS	0.5	33.33	0.83	4.59	8.23	**9.39**	5.19	4.71
S21S15	100×100×3 SHS	50×50×2 SHS	0.5	33.33	0.67	3.14	6.99	**7.93**	4.50	3.62
S21S16	100×100×3 SHS	50×50×1.6 SHS	0.5	33.33	0.53	3.52	5.87	**6.61**	3.84	4.09
S21S17	100×100×3 SHS	75×75×3 SHS	0.75	33.33	1.0	**5.98**	0.11	1.06	3.87	4.47
S21S18	100×100×3 SHS	75×75×2.5 SHS	0.75	33.33	0.83	**6.37**	0.08	1.06	3.61	5.29
S21S19	100×100×3 SHS	75×75×2 SHS	0.75	33.33	0.67	**4.26**	0.06	1.01	3.13	4.14
S21S20	100×100×3 SHS	75×75×1.6 SHS	0.75	33.33	0.53	4.55	0.04	0.92	2.49	**4.69**
S21S21	100×100×3 SHS	100×100×3 SHS	1	33.33	1.0	2.83	2.14	2.67	2.18	**4.25**
S21S22	100×100×3 SHS	100×100×2.5 SHS	1	33.33	0.83	2.90	1.79	2.21	1.76	**4.26**
S21S23	100×100×3 SHS	100×100×2 SHS	1	33.33	0.67	2.05	1.41	1.73	1.30	**3.08**
S21S24	100×100×3 SHS	100×100×1.6 SHS	1	33.33	0.53	2.09	1.12	1.38	1.01	**3.06**
S25S1	100×100×4 SHS	25×25×3 SHS	0.25	25	0.75	1.14	1.54	**2.25**	2.01	1.72
S25S2	100×100×4 SHS	25×25×2.5 SHS	0.25	25	0.63	1.34	1.44	**2.06**	1.84	1.92
S25S2	100×100×4 SHS	25×25×2 SHS	0.25	25	0.5	1.02	1.29	**1.77**	1.60	1.34
S25S4	100×100×4 SHS	25×25×1.6 SHS	0.25	25	0.4	1.03	1.11	**1.51**	1.39	1.28
S25S5	100×100×4 SHS	35×35×3 SHS	0.35	25	0.75	2.05	2.98	**3.97**	2.78	2.49
S25S6	100×100×4 SHS	35×35×2.5 SHS	0.35	25	0.63	2.33	2.67	**3.52**	2.50	2.83
S25S7	100×100×4 SHS	35×35×2 SHS	0.35	25	0.5	1.66	2.30	**2.98**	2.16	2.23
S25S8	100×100×4 SHS	35×35×1.6 SHS	0.35	25	0.4	1.87	1.94	**2.48**	1.84	2.33
S25S9	100×100×4 SHS	40×40×3 SHS	0.4	25	0.75	2.53	3.67	**4.59**	3.04	2.88
S25S10	100×100×4 SHS	40×40×2.5 SHS	0.4	25	0.63	2.87	3.26	**4.05**	2.73	3.30
S25S11	100×100×4 SHS	40×40×2 SHS	0.4	25	0.5	2.02	2.78	**3.41**	2.34	2.57
S25S12	100×100×4 SHS	40×40×1.6 SHS	0.4	25	0.4	2.26	2.32	**2.84**	1.98	2.79
S25S13	100×100×4 SHS	50×50×3 SHS	0.5	25	0.75	3.47	**4.95**	**4.95**	3.25	3.53
S25S14	100×100×4 SHS	50×50×2.5 SHS	0.5	25	0.63	3.85	4.31	**4.39**	2.89	4.06
S25S15	100×100×4 SHS	50×50×2 SHS	0.5	25	0.5	2.69	3.59	**3.70**	2.46	3.14
S25S16	100×100×4 SHS	50×50×1.6 SHS	0.5	25	0.4	2.96	2.93	3.05	2.05	**3.45**
S25S17	100×100×4 SHS	75×75×3 SHS	0.75	25	0.75	**4.03**	2.54	2.41	3.29	3.10
S25S18	100×100×4 SHS	75×75×2.5 SHS	0.75	25	0.63	**4.28**	2.22	2.47	2.88	3.54
S25S19	100×100×4 SHS	75×75×2 SHS	0.75	25	0.5	**3.10**	1.90	2.16	2.36	2.95
S25S20	100×100×4 SHS	75×75×1.6 SHS	0.75	25	0.4	**3.27**	1.60	1.79	1.89	3.26
S25S21	100×100×4 SHS	100×100×3 SHS	1	25	0.75	2.91	1.54	2.26	1.14	**4.03**
S25S22	100×100×4 SHS	100×100×2.5 SHS	1	25	0.63	3.51	0.83	1.22	0.91	**4.33**
S25S23	100×100×4 SHS	100×100×2 SHS	1	25	0.5	2.43	0.62	0.91	0.69	**3.10**
S25S24	100×100×4 SHS	100×100×1.6 SHS	1	25	0.4	2.42	0.44	0.65	0.52	**3.06**
S26S2	100×100×5SHS	25×25×2.5 SHS	0.25	20	0.5	1.18	0.85	1.35	1.21	**1.78**
S26S6	100×100×5SHS	35×35×2.5 SHS	0.35	20	0.5	2.03	1.56	2.17	1.68	**2.49**
S26S10	100×100×5SHS	40×40×2.5 SHS	0.4	20	0.5	2.48	1.90	2.52	1.83	**2.89**
S26S14	100×100×5SHS	50×50×2.5 SHS	0.5	20	0.5	3.33	2.43	2.82	1.95	**3.58**
S26S18	100×100×5SHS	75×75×2.5 SHS	0.75	20	0.5	**3.82**	1.24	1.62	1.94	3.32
S26S22	100×100×5SHS	100×100×2.5 SHS	1	20	0.5	2.26	0.36	0.51	0.82	**2.85**
S27S1	100×100×6SHS	25×25×3 SHS	0.25	16.67	0.5	0.92	0.59	1.01	0.99	**1.39**
S27S5	100×100×6SHS	35×35×3 SHS	0.35	16.67	0.5	1.60	1.12	1.67	1.39	**2.03**
S27S9	100×100×6SHS	40×40×3 SHS	0.4	16.67	0.5	1.95	1.37	1.96	1.52	**2.32**
S27S13	100×100×6SHS	50×50×3 SHS	0.5	16.67	0.5	2.65	1.75	2.21	1.64	**2.86**
S27S17	100×100×6SHS	75×75×3 SHS	0.75	16.67	0.5	**3.26**	0.95	1.18	1.77	2.82
S27S21	100×100×6SHS	100×100×3 SHS	1	16.67	0.5	2.06	0.34	0.48	0.66	**2.67**

**Table 11:** Influence of β on peak SCFs.

β	2γ = 33.33 τ = 1	2γ = 33.33 τ = 0.8	2γ = 33.33 τ = 0.67	2γ = 33.33 τ = 0.5	2γ = 20 τ = 0.5	2γ = 16.67 τ = 0.5	2γ = 25 τ = 0.75	2γ = 25 τ = 0.63	2γ = 25 τ = 0.5	2γ = 25 τ = 0.4
0.25	2.28	1.98	1.58	1.3	1.78	1.39	2.25	2.06	1.77	1.51
0.35	6.4	5.7	4.85	4.06	2.49	2.03	3.97	3.52	2.98	2.48
0.4	8.25	7.31	6.17	5.16	2.89	2.32	4.59	4.05	3.41	2.84
0.5	10.59	9.39	7.93	6.61	3.58	2.86	4.95	4.39	3.7	3.45
0.75	5.98	6.37	4.26	4.69	3.82	3.26	4.03	4.28	3.1	3.27
1	4.25	4.26	3.08	3.06	2.85	2.67	4.03	4.33	3.1	3.06

**Table 12:** Influence of τ on peak SCFs.

	**τ = 0.53**	**τ = 0.67**	**τ = 0.83**	**τ = 1**
β = 0.25, 2γ = 33.33	1.3	1.58	1.98	2.28
β = 0.35, 2γ = 33.33	4.06	4.85	5.7	6.4
β = 0.4, 2γ = 33.33	5.16	6.17	7.31	8.25
β = 0.5, 2γ = 33.33	6.61	7.93	9.39	10.59
β = 0.75, 2γ = 33.33	4.69	4.26	6.37	5.98
β = 1, 2γ = 33.33	3.06	3.08	4.26	4.25
	**τ = 0.40**	**τ = 0.50**	**τ = 0.63**	**τ = 0.75**
β = 0.25, 2γ = 25	1.51	1.77	2.06	2.25
β = 0.35, 2γ = 25	2.48	2.98	3.52	3.97
β = 0.4, 2γ = 25	2.84	3.41	4.05	4.59
β = 0.5, 2γ = 25	3.45	3.7	4.39	4.95
β = 0.75, 2γ = 25	3.27	3.1	4.28	4.03
β = 1, 2γ = 25	3.06	3.1	4.33	4.03

**Table 13:** Influence of 2γ on maximum SCFs.

2γ	β = 0.25 τ = 0.5	β = 0.35 τ = 0.5	β = 0.4 τ = 0.5	β = 0.5 τ = 0.5	β = 0.75 τ = 0.5	β = 1 τ = 0.5
33.33	1.3	4.06	5.16	6.61	4.69	3.06
25	1.77	2.98	3.41	3.7	3.1	3.1
20	1.78	2.49	2.89	3.58	3.82	2.85
16.67	1.39	2.03	2.32	2.86	3.26	2.67

**Table 14:** Constants *a*–*h*.

Constants	Line A	Line B	Line C	Line D	Line E	Peak SCF
*a*	5.683849	0.006668	0.069071	0.067958	4.584246	0.233257
*b*	−11.161296	−0.014833	−0.157049	−0.179585	−9.546303	−0.467340
*c*	5.662125	0.009966	0.101481	0.139751	5.075972	0.258524
*d*	−0.004096	−0.000034	−0.000232	−0.000231	−0.002281	−0.000598
*e*	−0.856986	0.465188	−0.051707	0.027163	−0.630846	0.096247
*f*	2.760551	7.327060	6.575175	6.553876	2.298098	3.977695
*g*	−0.737920	−5.509643	−4.793702	−5.285170	−0.338629	−2.104667
*h*	0.455557	0.736167	0.750481	0.682011	0.246473	0.700215

**Table 15:** Peak SCFs from predicted design equation with FEA.

Series Name	Non-Dimensional Parameters			SCF_FE_ of Concrete-Filled T-Joints under In-Plane Bending					Peak SCF		$\frac{{\mathrm{SCF}}_{\mathrm{Predicted}}}{{\mathrm{SCF}}_{\mathrm{FE}}}$
	β	2γ	τ	*A*	*B*	*C*	*D*	*E*	FE	Predicted	
**S21S1**	0.25	33.33	1	1.35	1.87	1.91	**2.28**	1.82	2.28	3.25	1.43
S21S2	0.25	33.33	0.83	1.59	1.59	1.65	1.95	**1.98**	1.98	2.86	1.44
S21S3	0.25	33.33	0.67	1.32	1.33	1.35	**1.58**	1.39	1.58	2.46	1.56
S21S4	0.25	33.33	0.53	0.95	1.1	1.11	**1.3**	1.23	1.3	2.09	1.60
S21S5	0.35	33.33	1	2.36	6.17	**6.4**	5.45	2.77	6.4	6.09	0.95
S21S6	0.35	33.33	0.83	2.66	5.62	**5.7**	4.96	3.21	5.7	5.35	0.94
S21S7	0.35	33.33	0.67	1.87	4.8	**4.85**	4.32	2.49	4.85	4.60	0.95
S21S8	0.35	33.33	0.53	2.06	4.02	**4.06**	3.7	2.41	4.06	3.91	0.96
S21S9	0.4	33.33	1	2.95	7.73	**8.25**	5.73	3.25	8.25	7.72	0.94
S21S10	0.4	33.33	0.83	3.34	6.86	**7.31**	5.22	3.79	7.31	6.78	0.93
S21S11	0.4	33.33	0.67	2.31	5.83	**6.17**	4.57	2.94	6.17	5.83	0.95
S21S12	0.4	33.33	0.53	2.6	4.88	**5.16**	3.93	3.09	5.16	4.95	0.96
S21S13	0.5	33.33	1	4.12	9.29	**10.59**	5.73	4	10.59	10.48	0.99
S21S14	0.5	33.33	0.83	4.59	8.23	**9.39**	5.19	4.71	9.39	9.20	0.98
S21S15	0.5	33.33	0.67	3.14	6.99	**7.93**	4.5	3.62	7.93	7.92	1.00
S21S16	0.5	33.33	0.53	3.52	5.87	**6.61**	3.84	4.09	6.61	6.72	1.02
S21S17	0.75	33.33	1	**5.98**	0.11	1.06	3.87	4.47	5.98	6.35	1.06
S21S18	0.75	33.33	0.83	**6.37**	0.08	1.06	3.61	5.29	6.37	5.57	0.87
S21S19	0.75	33.33	0.67	**4.26**	0.06	1.01	3.13	4.14	4.26	4.80	1.13
S21S20	0.75	33.33	0.53	4.55	0.04	0.92	2.49	**4.69**	4.69	4.07	0.87
S21S21	1	33.33	1	2.83	2.14	2.67	2.18	**4.25**	4.25	4.50	1.06
S21S22	1	33.33	0.83	2.9	1.79	2.21	1.76	**4.26**	4.26	3.95	0.93
S21S23	1	33.33	0.67	2.05	1.41	1.73	1.3	**3.08**	3.08	3.40	1.10
S21S24	1	33.33	0.53	2.09	1.12	1.38	1.01	**3.06**	3.06	2.88	0.94
S25S1	0.25	25	0.75	1.14	1.54	**2.25**	2.01	1.72	2.25	2.11	0.94
S25S2	0.25	25	0.63	1.34	1.44	**2.06**	1.84	1.92	2.06	1.87	0.91
S25S2	0.25	25	0.5	1.02	1.29	**1.77**	1.6	1.34	1.77	1.59	0.90
S25S4	0.25	25	0.4	1.03	1.11	**1.51**	1.39	1.28	1.51	1.36	0.90
S25S5	0.35	25	0.75	2.05	2.98	**3.97**	2.78	2.49	3.97	3.71	0.93
S25S6	0.35	25	0.63	2.33	2.67	**3.52**	2.5	2.83	3.52	3.28	0.93
S25S7	0.35	25	0.5	1.66	2.3	**2.98**	2.16	2.23	2.98	2.79	0.94
S25S8	0.35	25	0.4	1.87	1.94	**2.48**	1.84	2.33	2.48	2.39	0.96
S25S9	0.4	25	0.75	2.53	3.67	**4.59**	3.04	2.88	4.59	4.60	1.00
S25S10	0.4	25	0.63	2.87	3.26	**4.05**	2.73	3.3	4.05	4.07	1.00
S25S11	0.4	25	0.5	2.02	2.78	**3.41**	2.34	2.57	3.41	3.46	1.01
S25S12	0.4	25	0.4	2.26	2.32	**2.84**	1.98	2.79	2.84	2.96	1.04
S25S13	0.5	25	0.75	3.47	**4.95**	**4.95**	3.25	3.53	4.95	6.09	1.23
S25S14	0.5	25	0.63	3.85	4.31	**4.39**	2.89	4.06	4.39	5.39	1.23
S25S15	0.5	25	0.5	2.69	3.59	**3.7**	2.46	3.14	3.7	4.58	1.24
S25S16	0.5	25	0.4	2.96	2.93	3.05	2.05	**3.45**	3.45	3.92	1.14
S25S17	0.75	25	0.75	**4.03**	2.54	2.41	3.29	3.1	4.03	4.83	1.20
S25S18	0.75	25	0.63	**4.28**	2.22	2.47	2.88	3.54	4.28	4.27	1.00
S25S19	0.75	25	0.5	**3.1**	1.9	2.16	2.36	2.95	3.1	3.63	1.17
S25S20	0.75	25	0.4	**3.27**	1.6	1.79	1.89	3.26	3.27	3.11	0.95
S25S21	1	25	0.75	2.91	1.54	2.26	1.14	**4.03**	4.03	4.39	1.09
S25S22	1	25	0.63	3.51	0.83	1.22	0.91	**4.33**	4.33	3.89	0.90
S25S23	1	25	0.5	2.43	0.62	0.91	0.69	**3.1**	3.1	3.31	1.07
S25S24	1	25	0.4	2.42	0.44	0.65	0.52	**3.06**	3.06	2.83	0.92
S26S2	0.25	20	0.5	1.18	0.85	1.35	1.21	**1.78**	1.78	1.31	0.74
S26S6	0.35	20	0.5	2.03	1.56	2.17	1.68	**2.49**	2.49	2.20	0.88
S26S10	0.4	20	0.5	2.48	1.9	2.52	1.83	**2.89**	2.89	2.66	0.92
S26S14	0.5	20	0.5	3.33	2.43	2.82	1.95	**3.58**	3.58	3.43	0.96
S26S18	0.75	20	0.5	**3.82**	1.24	1.62	1.94	3.32	3.82	2.92	0.76
S26S22	1	20	0.5	2.26	0.36	0.51	0.82	**2.85**	2.85	2.80	0.98
S27S1	0.25	16.67	0.5	0.92	0.59	1.01	0.99	**1.39**	1.39	1.12	0.81
S27S5	0.35	16.67	0.5	1.6	1.12	1.67	1.39	**2.03**	2.03	1.79	0.88
S27S9	0.4	16.67	0.5	1.95	1.37	1.96	1.52	**2.32**	2.32	2.14	0.92
S27S13	0.5	16.67	0.5	2.65	1.75	2.21	1.64	**2.86**	2.86	2.68	0.94
S27S17	0.75	16.67	0.5	**3.26**	0.95	1.18	1.77	2.82	3.26	2.32	0.71
S27S21	1	16.67	0.5	2.06	0.34	0.48	0.66	**2.67**	2.67	2.27	0.85
STDEV											0.17
Mean											1.01
COV											0.17

**Table 16:** SCFs from predicted design equations and FEA at lines A–E.

Series Name	SCF_FE_					SCF_Predicted_					SCF_Predicted_/SCF_FE_				
	A	B	C	D	E	A	B	C	D	E	A	B	C	D	E
S21S1	1.35	1.87	1.91	**2.28**	1.82	1.47	2.30	2.64	2.60	1.86	1.09	1.23	1.38	1.14	1.02
S21S2	1.59	1.59	1.65	1.95	**1.98**	1.35	2.01	2.30	2.29	1.78	0.85	1.26	1.39	1.18	0.90
S21S3	1.32	1.33	1.35	**1.58**	1.39	1.23	1.72	1.96	1.98	1.68	0.93	1.29	1.45	1.25	1.21
S21S4	0.95	1.1	1.11	**1.3**	1.23	1.10	1.44	1.64	1.69	1.59	1.16	1.31	1.48	1.30	1.29
S21S5	2.36	6.17	**6.4**	5.45	2.77	2.49	6.03	6.39	5.14	2.84	1.06	0.98	1.00	0.94	1.03
S21S6	2.66	5.62	**5.7**	4.96	3.21	2.29	5.25	5.56	4.52	2.71	0.86	0.93	0.98	0.91	0.85
S21S7	1.87	4.8	**4.85**	4.32	2.49	2.08	4.49	4.73	3.91	2.57	1.11	0.94	0.98	0.90	1.03
S21S8	2.06	4.02	**4.06**	3.7	2.41	1.87	3.78	3.97	3.33	2.43	0.91	0.94	0.98	0.90	1.01
S21S9	2.95	7.73	**8.25**	5.73	3.25	3.13	8.07	8.46	6.01	3.41	1.06	1.04	1.03	1.05	1.05
S21S10	3.34	6.86	**7.31**	5.22	3.79	2.87	7.04	7.36	5.29	3.26	0.86	1.03	1.01	1.01	0.86
S21S11	2.31	5.83	**6.17**	4.57	2.94	2.61	6.01	6.26	4.57	3.09	1.13	1.03	1.02	1.00	1.05
S21S12	2.6	4.88	**5.16**	3.93	3.09	2.34	5.06	5.25	3.90	2.92	0.90	1.04	1.02	0.99	0.94
S21S13	4.12	9.29	**10.59**	5.73	4	4.53	9.44	10.37	5.65	4.59	1.10	1.02	0.98	0.99	1.15
S21S14	4.59	8.23	**9.39**	5.19	4.71	4.17	8.23	9.01	4.98	4.39	0.91	1.00	0.96	0.96	0.93
S21S15	3.14	6.99	**7.93**	4.5	3.62	3.78	7.03	7.68	4.30	4.16	1.20	1.01	0.97	0.96	1.15
S21S16	3.52	5.87	**6.61**	3.84	4.09	3.40	5.92	6.44	3.67	3.93	0.96	1.01	0.97	0.95	0.96
S21S17	**5.98**	0.11	1.06	3.87	4.47	5.94	0.36	1.34	4.17	4.82	0.99	3.29	1.26	1.08	1.08
S21S18	**6.37**	0.08	1.06	3.61	5.29	5.45	0.32	1.17	3.68	4.60	0.86	3.95	1.10	1.02	0.87
S21S19	**4.26**	0.06	1.01	3.13	4.14	4.95	0.27	0.99	3.18	4.37	1.16	4.49	0.98	1.02	1.05
S21S20	4.55	0.04	0.92	2.49	**4.69**	4.45	0.23	0.83	2.71	4.12	0.98	5.67	0.90	1.09	0.88
S21S21	2.83	2.14	2.67	2.18	**4.25**	2.87	2.00	2.49	1.92	4.00	1.01	0.93	0.93	0.88	0.94
S21S22	2.9	1.79	2.21	1.76	**4.26**	2.64	1.74	2.16	1.69	3.82	0.91	0.97	0.98	0.96	0.90
S21S23	2.05	1.41	1.73	1.3	**3.08**	2.39	1.49	1.84	1.46	3.62	1.17	1.06	1.06	1.12	1.18
S21S24	2.09	1.12	1.38	1.01	**3.06**	2.15	1.25	1.54	1.25	3.42	1.03	1.12	1.12	1.23	1.12
S25S1	1.14	1.54	**2.25**	2.01	1.72	1.39	1.19	1.57	1.57	1.78	1.22	0.77	0.70	0.78	1.04
S25S2	1.34	1.44	**2.06**	1.84	1.92	1.28	1.04	1.38	1.40	1.71	0.96	0.72	0.67	0.76	0.89
S25S2	1.02	1.29	**1.77**	1.6	1.34	1.16	0.88	1.16	1.19	1.61	1.13	0.68	0.65	0.75	1.20
S25S4	1.03	1.11	**1.51**	1.39	1.28	1.04	0.75	0.98	1.02	1.53	1.01	0.67	0.65	0.74	1.19
S25S5	2.05	2.98	**3.97**	2.78	2.49	2.21	2.93	3.52	2.95	2.58	1.08	0.98	0.89	1.06	1.03
S25S6	2.33	2.67	**3.52**	2.5	2.83	2.04	2.57	3.09	2.62	2.47	0.88	0.96	0.88	1.05	0.87
S25S7	1.66	2.3	**2.98**	2.16	2.23	1.84	2.17	2.60	2.24	2.33	1.11	0.94	0.87	1.04	1.05
S25S8	1.87	1.94	**2.48**	1.84	2.33	1.66	1.84	2.20	1.92	2.21	0.89	0.95	0.89	1.05	0.95
S25S9	2.53	3.67	**4.59**	3.04	2.88	2.69	3.92	4.58	3.46	3.01	1.06	1.07	1.00	1.14	1.05
S25S10	2.87	3.26	**4.05**	2.73	3.3	2.48	3.45	4.02	3.08	2.88	0.87	1.06	0.99	1.13	0.87
S25S11	2.02	2.78	**3.41**	2.34	2.57	2.24	2.91	3.38	2.63	2.72	1.11	1.05	0.99	1.12	1.06
S25S12	2.26	2.32	**2.84**	1.98	2.79	2.02	2.47	2.86	2.26	2.58	0.89	1.06	1.01	1.14	0.92
S25S13	3.47	**4.95**	**4.95**	3.25	3.53	3.70	5.07	5.75	3.56	3.85	1.07	1.02	1.16	1.10	1.09
S25S14	3.85	4.31	**4.39**	2.89	4.06	3.41	4.46	5.04	3.16	3.69	0.89	1.03	1.15	1.09	0.91
S25S15	2.69	3.59	**3.7**	2.46	3.14	3.07	3.76	4.24	2.70	3.48	1.14	1.05	1.15	1.10	1.11
S25S16	2.96	2.93	3.05	2.05	**3.45**	2.78	3.19	3.59	2.32	3.30	0.94	1.09	1.18	1.13	0.96
S25S17	**4.03**	2.54	2.41	3.29	3.1	4.53	2.42	2.33	2.84	3.79	1.12	0.95	0.97	0.86	1.22
S25S18	**4.28**	2.22	2.47	2.88	3.54	4.18	2.13	2.05	2.52	3.63	0.98	0.96	0.83	0.88	1.02
S25S19	**3.1**	1.9	2.16	2.36	2.95	3.77	1.80	1.72	2.16	3.43	1.22	0.94	0.80	0.91	1.16
S25S20	**3.27**	1.6	1.79	1.89	3.26	3.40	1.52	1.45	1.85	3.24	1.04	0.95	0.81	0.98	0.99
S25S21	2.91	1.54	2.26	1.14	**4.03**	3.08	1.19	1.63	1.19	3.82	1.06	0.78	0.72	1.04	0.95
S25S22	3.51	0.83	1.22	0.91	**4.33**	2.84	1.05	1.43	1.06	3.66	0.81	1.27	1.17	1.16	0.84
S25S23	2.43	0.62	0.91	0.69	**3.1**	2.56	0.89	1.20	0.90	3.45	1.05	1.43	1.32	1.31	1.11
S25S24	2.42	0.44	0.65	0.52	**3.06**	2.31	0.75	1.01	0.78	3.27	0.95	1.71	1.56	1.49	1.07
S26S2	1.18	0.85	1.35	1.21	**1.78**	1.22	0.60	0.90	0.93	1.65	1.03	0.71	0.67	0.76	0.93
S26S6	2.03	1.56	2.17	1.68	**2.49**	1.84	1.40	1.89	1.65	2.28	0.91	0.90	0.87	0.98	0.91
S26S10	2.48	1.9	2.52	1.83	**2.89**	2.19	1.85	2.41	1.92	2.61	0.88	0.97	0.96	1.05	0.90
S26S14	3.33	2.43	2.82	1.95	**3.58**	2.89	2.42	3.00	2.01	3.20	0.87	1.00	1.06	1.03	0.89
S26S18	**3.82**	1.24	1.62	1.94	3.32	3.32	1.49	1.53	1.65	2.95	0.87	1.20	0.95	0.85	0.89
S26S22	2.26	0.36	0.51	0.82	**2.85**	2.46	0.63	0.94	0.71	3.08	1.09	1.74	1.84	0.87	1.08
S27S1	0.92	0.59	1.01	0.99	**1.39**	1.27	0.44	0.73	0.75	1.68	1.38	0.75	0.72	0.75	1.21
S27S5	1.6	1.12	1.67	1.39	**2.03**	1.85	0.96	1.45	1.27	2.23	1.15	0.86	0.87	0.91	1.10
S27S9	1.95	1.37	1.96	1.52	**2.32**	2.16	1.25	1.81	1.46	2.51	1.11	0.91	0.92	0.96	1.08
S27S13	2.65	1.75	2.21	1.64	**2.86**	2.74	1.62	2.21	1.53	2.97	1.04	0.93	1.00	0.93	1.04
S27S17	**3.26**	0.95	1.18	1.77	2.82	2.96	1.10	1.24	1.28	2.58	0.91	1.15	1.05	0.72	0.91
S27S21	2.06	0.34	0.48	0.66	**2.67**	2.26	0.46	0.74	0.58	2.69	1.09	1.34	1.55	0.88	1.01
STDEV											0.12	0.89	0.24	0.15	0.11
Mean											1.02	1.25	1.02	1.01	1.02
COV											0.12	0.71	0.23	0.15	0.11

## Data Availability

Some or all data, models, or code that support the findings of this study are available from the corresponding author upon reasonable request.
